# Long-Term Outcomes in Thoracic Endovascular Aortic Repair for Complicated Type B Aortic Dissection or Intramural Hematoma Depending on Proximal Landing Zone

**DOI:** 10.3390/jcm12165380

**Published:** 2023-08-18

**Authors:** Philip Dueppers, Lorenz Meuli, Kerstin Stoklasa, Anna-Leonie Menges, Alexander Zimmermann, Benedikt Reutersberg

**Affiliations:** Department of Vascular Surgery, University Hospital Zurich, Rämistr. 100, 8091 Zurich, Switzerland; dr.dueppers@gmail.com (P.D.); lorenz.meuli@usz.ch (L.M.); kerstin.stoklasa@usz.ch (K.S.); anna-leonie.menges@usz.ch (A.-L.M.); alexander.zimmermann@usz.ch (A.Z.)

**Keywords:** TEVAR, type b aortic dissection, intramural hematoma, healthy landing zone, sizing, landing zone, Ishimaru zones, thoracic aorta

## Abstract

Thoracic endovascular aortic repair (TEVAR) is the preferred treatment for complicated type B aortic dissection (TBAD) or intramural hematoma (IMH). This study aimed to investigate the association of the proximal landing zone and its morphology with long-term outcomes in patients with TBAD or IMH. A total of 94 patients who underwent TEVAR for TBAD or IMH between 10/2003 and 01/2020 were included. The cohort was divided according to the proximal landing in Ishimaru zone 2 or 3 and the presence of a healthy landing zone (HLZ; non-dissected or aneurysmatic, ≥2 cm length). Primary outcome was freedom from aortic reintervention. Secondary endpoints were freedom from aortic growth, stroke, spinal cord ischemia, retrograde dissection, proximal stent-graft induced new entry (pSINE), debranching failure, and mortality. Outcomes were assessed using Cox proportional hazard models with mortality as a competing risk. A proximal TEVAR landing in zone 2 was associated with higher rates of reinterventions compared to zone 3 (33% vs. 15%, *p* = 0.031), spinal cord ischemia (8% vs. 0%, *p* = 0.037), and pSINE (13% vs. 2%, *p* = 0.032). No difference was found for the other outcomes, including mortality. Landing in dissected segments was not associated with impaired results. Proximal TEVAR landing in zone 3 may be preferable with regard to long-term aortic reintervention in patients with TBAD or IMH.

## 1. Introduction

Thoracic endovascular aortic repair (TEVAR) is the first-line therapy for the treatment of complicated acute type B aortic dissection (TBAD) or intramural hematoma (IMH) [1,2] and has replaced treatment by open surgery of the thoracic- or thoracoabdominal aorta [3].

In addition to the correct stent-graft selection and sizing, the proximal landing zone plays a crucial role in endovascular therapy [4,5,6]. The concept of TEVAR in TBAD or IMH is to cover the primary entry tear to induce false-lumen thrombosis and favorable aortic remodeling. However, there is paucity of data as to whether the proximal landing zone of the stent-graft must be in a non-dissected healthy aortic segment, or whether landing in a dissected segment is adequate in terms of long-term durability [7]. Due to the supra-aortic vessels, a sufficiently long healthy proximal landing zone is not always available without extension of the treatment into Ishimaru zone 2 [8]. Thus, adjunct procedures, such as open or endovascular supra-aortic debranching, are needed to preserve an antegrade perfusion of the left subclavian artery. Supra-aortic debranching brings a relevant risk of stroke and cervical nerve lesions, and extending the aortic covering into the aortic arch is associated with an increased risk for spinal cord ischemia (SCI) as well as retrograde aortic dissection, endoleaks, and aortic rupture [9,10]. Still, there is emerging evidence that a better long-term outcome can be achieved if the stent-graft is placed proximally in zone 2 rather than zone 3 [11,12]. It remains unclear if this is due to the length of the healthy landing zone or is more generally due to the location of the proximal stent-graft edge.

The aim of this study was therefore to investigate which has a more influence on the long-term outcome of patients with acute TBAD or IMH, a sufficiently long healthy landing zone or the aortic zone in which a proximal landing was obtained.

## 2. Materials and Methods

### 2.1. Patients

All patients treated with TEVAR for acute (<14 days from presentation) TBAD or IMH in the thoracic aorta from 1 October 2003 through 31 January 2020 at a single vascular center were included [1]. Patients with type A (TAAD) or non-A-non-B aortic dissection [13], isolated abdominal aortic dissections, as well as patients treated with elephant-trunk procedures or simultaneous visceral debranching were excluded. The institutional clinical information system was retrospectively screened and demographic, perioperative, and follow-up data were collected. Follow-up data were included up until 1 January 2021.

The Regional Review Board granted ethical approval and waived the requirement for written informed consent for retrospective analysis of their data (No. 2020-02236). However, all patients with a written refusal of institutional consent for retrospective analysis of their data had to be excluded.

### 2.2. Outcomes

Primary outcome was freedom from aortic reintervention. Secondary endpoints included freedom from aortic growth, stroke, SCI, retrograde TAAD, proximal stent-graft-induced new entry (pSINE, defined by Dong et al. as a new tear caused by the stent graft itself, excluding those created by natural disease progression or any iatrogenic injury from the endovascular manipulation [14]), debranching failure, and 30-day as well as overall mortality.

### 2.3. Definitions and Measurements

**Figure 1:**
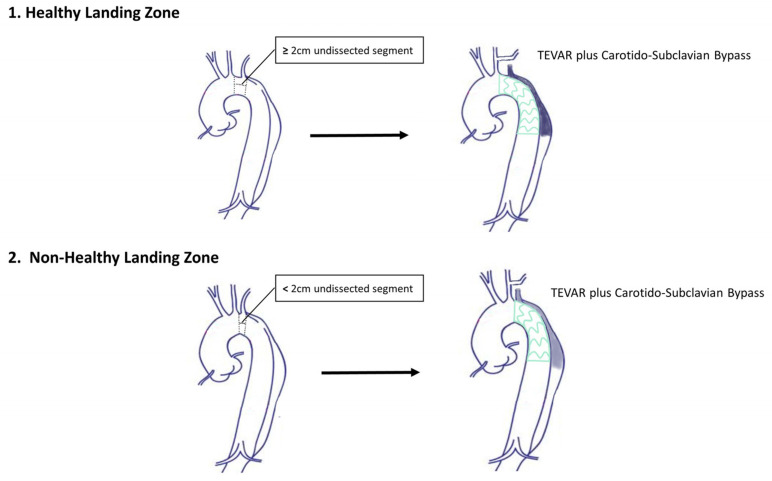
Definition of a healthy and a non-healthy proximal landing zone in an example of a type B aortic dissections where the thoracic stent graft was deployed proximally in Ishimaru Zone 2.

Aortic zones were classified according to Ishimaru (zone 2: aortic arch, distal to the left common carotid artery, including the origin of the left subclavian artery, zone 3: aortic arch, distal to the origin of the left subclavian artery) [8]. A healthy landing zone (HLZ) was defined as a non-dissected aortic segment with a length ≥20 mm as recommended for endovascular treatment of thoracic aortic aneurysms to achieve sufficient sealing (Figure 1) [7,11,15]. Aortic reinterventions included all thoracic aortic reinterventions related to endoleaks or persisting false lumen perfusion, rupture, or dissection propagation, as well as other procedures related to the original aortic pathology. Aortic growth was defined as an increase of ≥5 mm in maximal aortic diameter [15]. Debranching failure was defined as occlusion of any graft that was used for supra-aortic debranching.

Pre- and postoperative computed tomography angiography (CTA) scans were reviewed using a three-dimensional workstation (XERO Viewer 8.1.2, Agfa Healthcare N.V., Mortsel, Belgium) and a center line-based method for measurement. The proximal landing zone was retrospectively assessed based on the first postoperative CTA. Subsequently, its length was measured from the level of the future proximal end of the stent-graft to the beginning of the unhealthy segment in the preoperative CTA. The proximal landing zone was 0 mm in patients where the stent-graft landed in an unhealthy segment.

### 2.4. Statistical Analysis

Patient’s characteristics, procedural details, and outcomes are summarized and presented by tables stratified by the proximal stent-graft landing zone (Ishimaru zone 2 versus zone 3). The distribution of continuous data was visually inspected and formally tested using the Shapiro–Wilk test. Continuous variables were summarized by mean and standard deviation if normally distributed and compared using Student’s *t*-test. Skewed continuous variables were summarized by median and quartiles (Q1–Q3) and compared using the Mann–Whitney U test. Categorical variables were summarized with counts and percentages for each level of the variable and compared using Fisher’s exact test.

Freedom from reintervention and the other endpoints were analyzed using a Cox proportional hazard model with mortality as a competing risk. All variables that were associated with reintervention at *p* ≤ 0.2 in the overall multivariate analysis were included in the final model to elaborate on the association of the landing zone with the outcomes. Due to the low number of missing variables, complete case analyses were performed. The proportional hazard assumption was tested and verified using scaled Schoenfeld residuals for each variable in each model.

To allow inclusion of this data for further investigations, patient’s characteristics and procedural details were also stratified by the morphology of the proximal landing zone (healthy versus non-healthy) and presented in Supplementary Materials.

Analyses were conducted with R-Studio, R version 4.2.3, on MacOS version 12.5.1 or IBM SPSS Statistics Version 26 on Windows. All *p*-values are two-sided with an alpha-level of 5%, and no adjustment for multiple testing was performed.

## 3. Results

### 3.1. Patients and Procedural Details

A total of 94 patients at a median age of 70 (Q1–Q3: 59–78) years were identified. The majority of patients presented with TBAD (n = 84, 89%) and only ten patients (11%) presented with type B IMH. A total of 106 aortic stent-grafts (Gore TAG = 91, 86%) were implanted after a median time of 6 (1–13) days post-dissection (Table 1). In 39/94 patients (41%), the proximal landing zone was Ishimaru zone 2 or more proximal, whereas in 55/94 patients (59%), the landing zone was more distal (zone ≥ 3). The indications for TEVAR were organ malperfusion (33%), followed by expansion of the dissection (25%), aortic rupture (18%), recurrent pain (18%), and refractory hypertension (7%), and did not differ between Ishimaru zones (Table 2). However, there was a tendency towards a more extensive proximal aortic covering, i.e., TEVAR landing in zone 2 rather than 3, in patients with malperfusion (38% vs. 20%) and aortic ruptures (26% vs. 20%), *p* = 0.145.

Patients treated with TEVAR in zone 2 were significantly more likely to have a healthy proximal landing zone ≥20 mm than patients receiving TEVAR in zone 3 and further distally, 79% vs. 56%, respectively, *p* = 0.02 (Table 2). Of note: In 32 patients (34%), a HLZ of ≥20 mm distal to the left subclavian artery (LSA) was already present at baseline. In 22 patients (23%), the LSA was debranched with carotid-subclavian or carotid-axillary bypasses (n = 14) or with parallel grafts (n = 8). In another 17 patients (18%), the LSA was either partially (n = 8) or completely (n = 9) covered by the thoracic stent-graft without debranching.

However, this created a proximal landing zone of at least 20 mm in only 31 patients. In three patients after debranching, two patients after parallel grafts, and three patients without debranching and complete LSA covering, the HLZ remained <20 mm. These eight patients were counted as non-HLZ. Detailed data on demographics and interventions stratified by Ishimaru zone are presented in Table 1 and Table 2 and stratified by HLZ in Supplementary Tables S1 and S2.

### 3.2. Aortic Reinterventions

**Figure 2:**
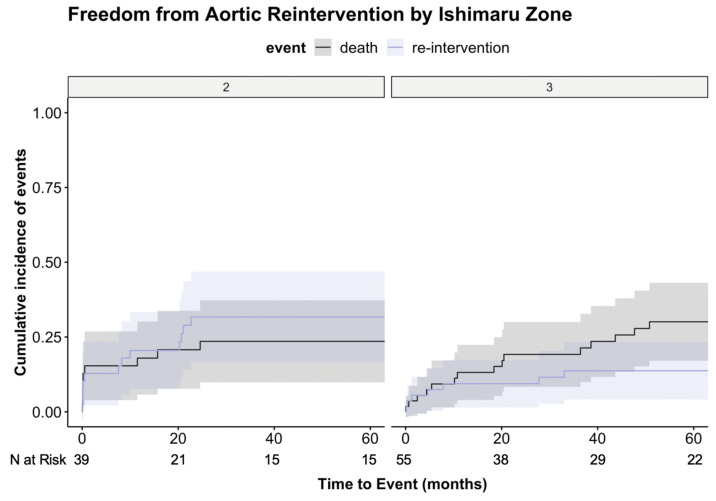
Cumulative incidence of both events (reintervention and mortality) visualizing the competing risk analysis for the proximal landing zone depending on Ishimaru zone 2 vs. zone 3. Death as a competing risk for aortic events occurred throughout the entire study period in both groups. *p* = 0.038 (re-intervention); *p* = 0.359 (death).

Median follow-up was 56 (17–113) months. The overall aortic reintervention rate was 22% (n = 21) and included distal stent-graft extension (n = 11), proximal extension (n = 9), and, in one case, stent-graft relining due to a type III endoleak. The unadjusted cumulative incidence of aortic reinterventions at 1 year after TEVAR was 21% in zone 2 and 9% after TEVAR in zone 3, and at 5 years, 32% versus 14%, respectively, *p* = 0.038 (Figure 2). Table 3 shows the clinical and morphological outcomes by Ishimaru zone. Six of the 13 reinterventions for patients treated in zone ≤ 2 required a proximal extension due to an endoleak type IA, where only three patients initially treated in zone 3 needed a proximal extension. Out of the six patients with initial implantation up to zone 2 who required proximal extension during follow up, two were treated with a single branched endoprosthesis (NEXUS, Endospan, Hertsliya, Israel) combined with either a carotido-carotid-subclavian bypass or a parallel graft for the left common carotid artery. Additionally, one patient received a Frozen Elephant Trunk implantation, another underwent open ascending and arch replacement, and two others underwent TEVAR extension with carotido-carotido-subclavian bypass. There were also more patients initially treated in zone 2 who required a distal stent-graft extension (22% vs. 7%).

**Figure 3:**
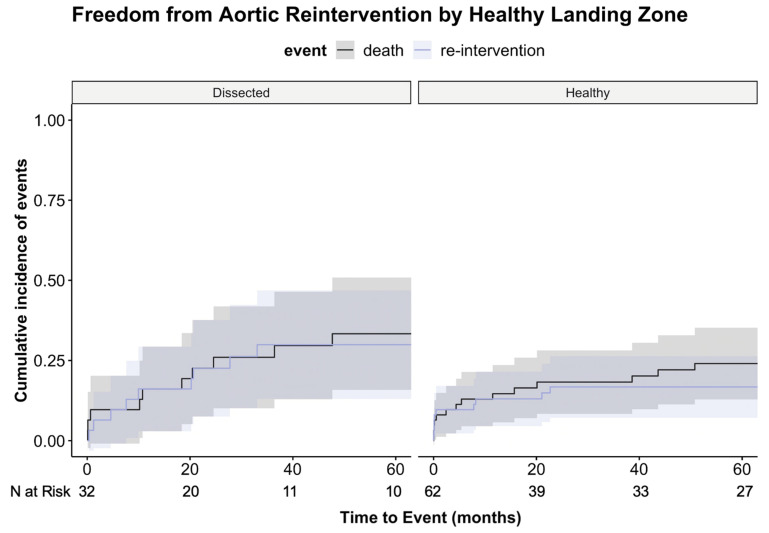
Cumulative incidence of both events (reintervention and mortality) visualizing the competing risk analysis for a healthy vs. a non-healthy (dissected) proximal landing zone. Death as a competing risk for aortic events occurred throughout the entire study period in both groups. *p* = 0.187 (re-intervention); *p* = 0.668 (death).

The unadjusted cumulative incidence of aortic reinterventions at 1 year after TEVAR was 16% in patients with a dissected landing zone and 13% after TEVAR in patients with a healthy landing zone; at 5 years, the incidence was 30% versus 17%, respectively, *p* = 0.187 (Figure 3).

**Figure 4:**
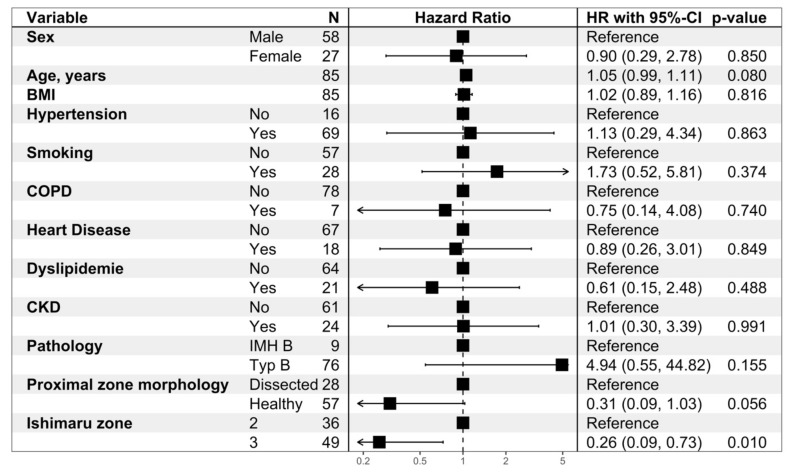
Multivariable Cox proportional hazard model for aortic re-interventions with death as competing risk. Diabetes was excluded from this analysis due to statistical separation. The proportional hazard assumption was tested and hold for all the variables and the entire model.

Figure 4 shows the results of the multivariable Cox proportional hazard model for aortic reintervention with mortality as a competing risk. The observed association of Ishimaru zone with aortic reintervention remained significant in the adjusted analysis. Patients with a proximal TEVAR landing in zone 3 had a significantly better long-term aortic reintervention free survival compared to zone 2, hazard ratio (HR) 0.26 (95%-CI: 0.09 to 0.73) *p* = 0.010. The same model showed a non-significant tendency towards better long-term outcome in patients with a HLZ, HR 0.31 (95%-CI: 0.09 to 1.03, *p* = 0.056).

### 3.3. Secondary Outcomes

Aortic growth was observed in 12 patients after a median time of 2.2 years (0.8, 6.0) with a mean diameter increase of 9 mm (SD: 7 mm). The incidence of aortic growth was higher in patients with proximal landing in zone 2, than in zone 3 (n = 6, 16% vs. n = 6, 11%, respectively), as well as a non-HLZ (n = 5, 16%) compared to a HLZ (n = 7, 11%), but this was not statistically significant (*p* = 0.459 and *p* = 0.531, respectively) (Table 3 and Supplementary Table S3).

Significantly more patients with TEVAR in zone 2 suffered from SCI (8% vs. 0%, *p* = 0.037), as well as from pSINE (13% vs. 2%, *p* = 0.032). No significant differences were found for the other secondary outcomes, especially stroke, which was 8% vs. 4%, respectively, *p* = 0.388. Concerning retrograde TAAD, a tendency towards more cases in zone 2 than when landing in zone 3 was observed (10% vs. 2%, respectively, *p* = 0.072) (Table 3).

Comparing HLZ vs. non-HLZ, no statistically significant differences were observed for stroke (3% HLZ vs. 9% non-HLZ, *p* = 0.208), SCI (5% HLZ vs. 0% non-HLZ, *p* = 0.200), or retrograde TAAD (5% HLZ vs. 6% non-HLZ, *p* = 1.000). Follow-up CTA revealed six patients with pSINE (8% HLZ vs. 3% non-HLZ, *p* = 0.606) and one debranching failure (2% HLZ vs. 0% non-HLZ, *p* = 1.000). The latter comprised a patient with an asymptomatic carotid-axillary bypass occlusion (Supplementary Table S3).

Stent-graft migration was not observed in any patient (Table 3). Regarding patients with stent-graft coverage of the LSA, one stroke after complete and one pSINE after partial coverage was observed, respectively. Neither retrograde TAAD nor SCI occurred in these patients.

The 30-day mortality was 10% (n = 9) and equally distributed, both when comparing by zone (zone 2, 9% vs. zone 3, 10%, *p* = 0.809), as well as in relation to a HLZ (non-HLZ, 9% vs. HLZ, 10%, *p* = 1.00) (Table 3 and Supplementary Table S3). The nine deaths resulted from various causes, including one instance of common iliac artery rupture leading to septic multi-organ failure, one retrograde TAAD, two ruptures related to TBAD, one case of sepsis, one unknown cause of death that occurred after transfer to another hospital, one mesenteric ischemia, one case with treatment limitations due to hypoxic encephalopathy, and one intracerebral hemorrhage following oral therapeutic anticoagulation.

## 4. Discussion

This study demonstrated that, in most patients who received a TEVAR for acute complicated TBAD or IMH, the proximal landing was in zone 3 (59%), and that, in most cases, a HLZ of at least 20 mm (66%) could be achieved. Landing in zone 2 was associated with impaired long-term outcomes, especially with significantly more aortic reinterventions. Overall, this study suggests that the choice of the proximal landing zone with respect to the Ishimaru zones seems to influence the outcome more than whether it is a HLZ or non-HLZ.

The reason that significantly more patients required reinterventions when landing in zone 2 in the long-term could be that debranching procedures cannot always create a true HLZ, either through parallel grafts with gutterleaks or endoleaks, or through bypass or LSA transposition/bypass [16,17]. Generally, in many cases, a stump of the LSA remains, which could be considered a potential weak point for a type IA endoleak. Sometimes the proximal tip of the TEVAR prosthesis slips into this stump, which then requires further proximal extension with additional debranching of the LCCA, if necessary. Newer technologies, such as single branched TEVAR stent-grafts with the branch integrated into the prosthesis (such as the Castor prosthesis, MicroPort Endovascular MedTech Co., Ltd., Shanghai, China) [18], or in situ fenestration plus bridging stent-grafts could provide a remedy for this [19], as complete extension of the HLZ might be achieved in this way. However, this needs to be investigated in the future. Supra-aortic debranching was generally performed to extend the landing to zone 2 with good results in terms of vessel patency. However, debranching was associated with relevant complications, such as significantly more SCIs and pSINE, as well as a tendency towards more retrograde TAAD. Interpretation of these findings is difficult as patients requiring debranching generally have a disease that already involves a more proximal segment of the aorta which itself might be associated with an increased risk for retrograde TAAD and other complications. However, these findings underline the caution that is needed when planning and conducting TEVAR in patients with TBAD or IMH. In addition, we observed a tendency towards more strokes in the patients with proximal landing in zone 2, but this difference was not statistically significant.

Our study findings contradict previous findings by Mesar et al. and Kuo et al., who both showed better outcomes for zone 2 TEVAR compared to zone 3 TEVAR. However, Mesar et al. focused solely on comparing zones 2 and 3, without considering the length of the HLZ. The findings revealed that, when landing in zone 2 instead of zone 3, there was a reduced need for reintervention [11]. However, the analyzed patients were substantially different from our cohort in terms of HLZ. Kuo et al. and Mesar et al. observed a HLZ of at least 20 mm in only 16.9% and 10.5%, respectively [7,11]. Kuo et al. even described that, in 47.3% of patients, all supra-aortic vessels would have required coverage in order to achieve a HLZ of 20 mm. In our cohort, 34% of the patients already had a HLZ of 20 mm distal to the LSA at baseline. This demonstrates that, in a substantial proportion of the studied cohort, the pathology was more distal, hindering a fair comparison. Of note, Kuo et al. included not only acute aortic dissections, but also 20 chronic TBADs with aneurysmatic formation, which limits comparability [7].

Regarding the secondary outcomes, the stroke rate of 5% was in line with recently published data from the International Registry of Acute Aortic Dissections (IRAD), which reported a stroke rate of 4.6% for aortic dissections treated with TEVAR alone [20]. The rate also approximates the 4.8% reported by Mesar et al. The latter found no significant difference, although there were clinically meaningful differences between proximal landing zones. In patients with TEVAR in Ishimaru zone 2 with coverage of the LSA without revascularization, Mesar et al. reported a 10% stroke rate, while in the group with LSA revascularization, the stroke rate was 5.3%, which corresponds to an absolute risk reduction of almost 50%. In those where the stent-graft landed proximally in zone 3, the rate was only 2.9% [11]. In the current study, we observed a nearly 3-fold greater stroke rate in patients with proximal landing in a non-HLZ compared to patients with proximal landing in an HLZ. This clinically highly relevant finding was not statistically significant due to the low number of events. Due to the small sample size and the low number of events, there is a substantial risk for a type II error. This finding is potentially dramatic and needs further exploration in larger studies in the future. Of note, more patients in the HLZ group had a partial (HLZ, 18% vs. non-HLZ, 0%) or complete (HLZ, 10% vs. non-HLZ, 9%) LSA coverage without revascularization, and LSA coverage was not associated with a higher stroke rate. However, according to the current guidelines, revascularization of the LSA should be considered if it is intentionally covered to prevent neurological complications [2,21,22,23,24,25,26,27,28]. This also includes SCI. Although none of the patients in whom the LSA was covered suffered from SCI in the current study, great caution is mandatory as collaterals of the LSA can compensate for a TEVAR-related reduction in spinal cord perfusion. Of note: our study showed significantly more patients with SCI after TEVAR in zone 2 (8% vs. 0% in zone 3). The same was observed in the study by Mesar et al. They described an SCI rate of up to 20%, which was significantly higher in patients in whom the LSA was covered and not revascularized than in patients with revascularization (2.6%). Kuo and colleagues concluded in their analysis that supra-aortic debranching is recommended to safely land in a non-dissected segment (regardless of a minimum length), as this can reduce the rate of retrograde TAAD [7]. In general, this cannot be reproduced in the current study because, in HLZ patients, in whom more debranching was performed, the rate of retrograde dissections was only slightly lower (HLZ, 5% vs. non-HLZ, 6%) and this was statistically not significant. Further, when comparing by aortic zones, there was even a tendency towards more retrograde TAAD when the landing zone was in zone 2. This observation is consistent with the results of Mesar et al., who showed no statistically significant difference in the rate of retrograde TAAD depending on the landing zone (zone 2 vs. 3) and the need of debranching [11].

The 30-day mortality of 10% of the presented study cohort is within the range of mortality generally described for complicated TBAD, which has not yet changed despite the increase in endovascular therapy over the years [3,29,30]. Additionally, there was no significant difference in short- and long-term mortality between patients who landed proximally in zone 2 vs. zone 3 or a HLZ vs. non-HLZ. Thus, an initially more extensive procedure with supra-aortic debranching or an increase of the landing zone by partial or complete occlusion of the LSA was not associated with an increase in mortality.

The ideal and minimum length of the proximal landing zone in TEVAR for TBAD remains unknown but is of paramount interest. Different lengths are propagated without having been investigated on uniform collectives. Piazza et al. investigated the ideal landing zone depending on the arch configuration. In their analysis of 140 patients, they concluded that a proximal sealing length of 20 mm was only sufficient in patients with type I anatomy. They proposed aiming for a sealing length of at least 25 mm in patients with a type II or III aortic arch. However, this was a heterogeneous cohort of patients with different aortic pathologies (only 12 acute aortic dissections) [31]. Czerny et al. state in their expert consensus that the use of stent-grafts is not recommended in patients with a proximal and/or distal landing zone length of less than 25 mm. They even recommend a length of 30 mm for complete endovascular arch replacement regardless of pathology, although no data are available to support this [13].

### Strengths and Limitations

In addition to the limitations of the study already mentioned, the following should be highlighted: This was a retrospective single-center series that took place over a long observation period. During this period, different teams were involved, and different debranching methods were used (both endovascular and open surgery). However, in the latter, no significant differences were found between the two techniques in terms of reintervention rates and mortality, as our research group has recently shown [16]. There was no uniform standard during the long observation period for how and where the stent-grafts were deployed or how long the proximal landing zone should be. The decision was left to the discretion of the surgeons performing the procedures. Remodeling was not studied as an outcome measure, as recommended in the Society of Vascular Surgery (SVS) reporting standards, because Kuo et al. already showed that the length of the proximal landing zone does not affect subsequent aortic remodeling, but rather that the primary entry tear is sealed [7,15].

A strength of the study is that stent-grafts from the same manufacturer were used in almost 90% of patients, which reduces confounding due to different properties of the various stent-grafts on the market.

## 5. Conclusions

This study provides insights into the choice of proximal landing zone in TEVAR for acute complicated TBAD and IMH. Most patients had their stent-graft placed in Ishimaru zone 3 with a HLZ of at least 20 mm achieved. Within its limitations, the study suggests that a TEVAR landing in Ishimaru zone 2 may be associated with a higher aortic re-intervention rate compared to a TEVAR landing in zone 3. In this regard, the proximal extent of the TEVAR landing seems to have a greater impact on long-term outcomes than the morphology of the landing zone, i.e., healthy or dissected. Larger and prospective studies are urgently needed for this procedure, which is performed daily all over the world.

## Figures and Tables

**Table 1:** Demographics.

Characteristic	Total (n = 94)	Zone 2 (n = 39)	Zone 3 (n= 55)	*p*-Value
Age, years, median (Q1, Q3)	70 (59, 78)	70 (58, 76)	69 (60, 78)	0.803
Male sex	65 (69%)	27 (69%)	38 (69%)	0.988
Body mass index, mean (SD)	26 (5)	25 (4)	26 (5)	0.304
**Aortic disease**				
Type B aortic dissection	84 (89%)	90 (90%)	49 (89%)	0.300
Type B intramural hematoma	10 (11%)	4 (10%)	6 (11%)	
**Comorbidities**				
Arterial hypertension	57 (80%)	32 (82%)	43 (78%)	0.645
Nicotine abuse	31 (33%)	13 (33%)	18 (33%)	0.951
Chronic kidney disease	26 (28%)	10 (26%)	16 (29%)	0.713
Dyslipidemia	23 (24%)	12 (31%)	11 (20%)	0.231
Coronary heart disease	20 (21%)	7 (18%)	13 (24%)	0.507
Diabetes mellitus	9 (10%)	4 (10%)	5 (9%)	0.850
COPD	7 (7%)	3 (8%)	4 (7%)	0.939

COPD = chronic obstructive pulmonary disease; Q1 = quartile 1 (25%); Q3 = quartile 3 (75%); SD = Standard deviation.

**Table 2:** In-hospital and procedural data.

Characteristic	Total (n = 94)	Zone 2 (n = 39)	Zone 3 (n = 55)	*p*-Value
Length of stay, days, median (Q1, Q3)	13 (8, 19)	14 (8, 20)	12 (8,18)	0.301
Time to treatment, days, median (Q1, Q3)	6 (1, 13)	4 (0, 12)	8 (1, 12)	0.179
Indication for TEVAR, multiple per patient possible				0.145
Pain	18 (19%)	7 (18%)	11 (20%)	
Hypertension	5 (5%)	1 (3%)	4 (7%)	
Malperfusion	26 (28%)	15 (38%)	11 (20%)	
Expansion or Progression	24 (26%)	6 (15%)	18 (33%)	
Aortic Rupture	21 (22%)	10 (26%)	11 (20%)	
Stent-grafts used for TEVAR	106	47	59	n.a.
Gore	91	43	48	
Jotec	8	2	6	
Cook	5	2	3	
Medtronic	2	0	2	
Morphology of Proximal Landing Zone				0.020
HLZ	62 (66%)	31 (79%)	31 (56%)	
Non-HLZ	32 (34%)	8 (21%)	24 (44%)	
Length of HLZ, mm, median (Q1, Q3) Number of patients with no HLZ	21 (12, 29) 19 (20%)	23 (20, 31) 4 (10%)	20 (0, 27) 15 (27%)	0.022
LSA Management				
Carotid-axillary/subclavian bypass	14 (15%)	14 (36%)	0 (0%)	
Parallel graft	8 (9%)	8 (21%)	0 (0%)	
Partial coverage w/o debranching	8 (9%)	8 (21%)	0 (0%)	
Full coverage w/o debranching	9 (10%)	9 (23%)	0 (0%)	

HLZ = healthy landing zone; Q1 = quartile 1 (25%); Q3 = quartile 3 (75%); LSA = left subclavian artery; TEVAR = thoracic aortic endovascular repair, n.a.= not applicable.

**Table 3:** Clinical and morphological outcome.

Outcome	Total (n = 94)	Zone 2 (n = 39)	Zone 3 (n = 55)	*p*-Value
Follow-up, months, median (Q1, Q3)	53 (14, 111)	58 (13, 112)	51 (19, 108)	0.875
Aortic reintervention, overall	21 (22%)	13 (33%)	8 (15%)	0.038
Distal stent-graft extension	11 (12%)	7 (22%)	4 (7%)	
⚬ aneurysm growth	10 (11%)	7 (18%)	3 (5%)	
⚬ aortic rupture	1 (1%)	0 (0%)	1 (2%)	
Proximal extension (EL IA)	9 (10%)	6 (15%)	3 (5%)	
Relining of type III endoleak	1 (1%)	0 (0%)	1 (2%)	
Aortic growth (≥5 mm)	12 (13%)	6 (16%)	6 (11%)	0.459
Stroke	5 (5%)	3 (8%)	2 (4%)	0.388
Spinal cord ischemia	3 (3%)	3 (8%)	0 (0%)	0.037
Stent-graft migration	0 (0%)	0 (0%)	0 (0%)	n.a.
Debranching failure (carotid-subclavian or axillary bypass)	1 (1%)	1 (3%)	0 (0%)	0.233
Retrograde TAAD	5 (5%)	4 (10%)	1 (2%)	0.072
pSINE	6 (6%)	5 (13%)	1 (2%)	0.032
30-day mortality	9 (10%)	3 (9%)	6 (10%)	0.809
Overall mortality	42 (45%)	18 (46%)	24 (44)	0.809

EL IA = type IA endoleak; n.a. = not applicable; Q1 = quartile 1 (25%); Q3 = quartile 3 (75%); pSINE = proximal stent-graft induced new entry; TAAD = type A aortic dissection.

## Data Availability

The data presented in this study are available on request from the corresponding author. The data are not publicly available due to regional regulations.

## Supplementary Materials

The following supporting information can be downloaded at: https://www.mdpi.com/article/10.3390/jcm12165380/s1, Table S1: Demographics by healthy landing zone; Table S2: In-hospital and procedural data by healthy landing zone and Table S3: Clinical and morphological outcome by healthy landing zone.

## References

[1] Erbel R., Aboyans V., Boileau C., Bossone E., Bartolomeo R.D., Eggebrecht H., Evangelista A., Falk V., Frank H., Gaemperli O. (2014). 2014 ESC Guidelines on the Diagnosis and Treatment of Aortic diseasesDocument Covering Acute and Chronic Aortic Diseases of the Thoracic and Abdominal Aorta of the adultThe Task Force for the Diagnosis and Treatment of Aortic Diseases of the European Society of Cardiology (ESC). Eur. Heart J..

[2] Riambau V., Böckler D., Brunkwall J., Cao P., Chiesa R., Coppi G., Czerny M., Fraedrich G., Haulon S., Jacobs M.J. (2017). Management of Descending Thoracic Aorta Diseases. Eur. J. Vasc. Endovasc. Surg..

[3] Reutersberg B., Salvermoser M., Trenner M., Geisbüsch S., Zimmermann A., Eckstein H., Kuehnl A. (2019). Hospital Incidence and In-Hospital Mortality of Surgically and Interventionally Treated Aortic Dissections: Secondary Data Analysis of the Nationwide German Diagnosis-Related Group Statistics from 2006 to 2014. J. Am. Heart Assoc..

[4] Meuli L., Zimmermann A. (2021). Proximal Sealing Length in TEVAR: Dependent on Aortic Arch Type?. Eur. J. Vasc. Endovasc. Surg..

[5] Rychla M., Dueppers P., Meuli L., Rancic Z., Menges A.-L., Kopp R., Zimmermann A., Reutersberg B. (2022). Influence of Measurement and Sizing Techniques in Thoracic Endovascular Aortic Repair on Outcome in Acute Complicated Type B Aortic Dissections. Interact. CardioVascular Thorac. Surg..

[6] Xiang D., Chai B., Huang J., Liang H., Liang B., Zhao H., Zheng C. (2023). The Impact of Oversizing in Thoracic Endovascular Aortic Repair on Long-Term Outcomes in Uncomplicated Type B Aortic Dissection: A Single-Center Retrospective Study. J. Endovasc. Therapy.

[7] Kuo E.C., Veranyan N., Johnson C.E., Weaver F.A., Ham S.W., Rowe V.L., Fleischman F., Bowdish M., Han S.M. (2019). Impact of Proximal Seal Zone Length and Intramural Hematoma on Clinical Outcomes and Aortic Remodeling after Thoracic Endovascular Aortic Repair for Aortic Dissections. J. Vasc. Surg..

[8] Ishimaru S. (2004). Endografting of the Aortic Arch. J. Endovasc. Ther..

[9] Geisbüsch P., Kotelis D., Müller–Eschner M., Hyhlik-Dürr A., Böckler D. (2011). Complications after Aortic Arch Hybrid Repair. J. Vasc. Surg..

[10] Ockert S., Eckstein G., Lutz B., Reeps C., Eckstein H.-H. (2015). Aortic Hemiarch Hybrid Repair. J. Vasc. Surg..

[11] Mesar T., Alie-Cusson F.S., Rathore A., Dexter D.J., Stokes G.K., Panneton J.M. (2022). A More Proximal Landing Zone Is Preferred for Thoracic Endovascular Repair of Acute Type B Aortic Dissections. J. Vasc. Surg..

[12] Kudo T., Kuratani T., Sawa Y., Miyagawa S. (2023). Effectiveness of Proximal Landing Zone 1 and 2 Thoracic Endovascular Aortic Repair for Type B Aortic Dissection by Comparing Outcomes with Thoracic Arch Aneurysm. J. Endovasc. Ther..

[13] Czerny M., Pacini D., Aboyans V., Al-Attar N., Eggebrecht H., Evangelista A., Grabenwöger M., Stabile E., Kolowca M., Lescan M. (2021). Current Options and Recommendations for the Use of Thoracic Endovascular Aortic Repair in Acute and Chronic Thoracic Aortic Disease: An Expert Consensus Document of the European Society for Cardiology (ESC) Working Group of Cardiovascular Surgery, the ESC Working Group on Aorta and Peripheral Vascular Diseases, the European Association of Percutaneous Cardiovascular Interventions (EAPCI) of the ESC and the European Association for Cardio-Thoracic Surgery (EACTS). Eur. J. Cardio-Thorac. Surg..

[14] Dong Z., Fu W., Wang Y., Wang C., Yan Z., Guo D., Xu X., Chen B. (2010). Stent Graft-Induced New Entry after Endovascular Repair for Stanford Type B Aortic Dissection. J. Vasc. Surg..

[15] Lombardi J.V., Hughes G.C., Appoo J.J., Bavaria J.E., Beck A.W., Cambria R.P., Charlton-Ouw K., Eslami M.H., Kim K.M., Leshnower B.G. (2020). Society for Vascular Surgery (SVS) and Society of Thoracic Surgeons (STS) Reporting Standards for Type B Aortic Dissections. J. Vasc. Surg..

[16] Dueppers P., Meuli L., Reutersberg B., Hofmann M., Messmer F., Zimmermann A. (2022). Early and Mid-Term Outcomes of Open versus Endovascular Left Subclavian Artery Debranching for Thoracic Aortic Diseases. Ann. Thorac. Cardiovasc. Surg..

[17] Dueppers P., Reutersberg B., Rancic Z., Messmer F., Menges A.-L., Meuli L., Rychla M., Zimmermann A. (2022). Long-Term Results of Total Endovascular Repair of Arch-Involving Aortic Pathologies Using Parallel Grafts for Supra-Aortic Debranching. J. Vasc. Surg..

[18] Huang H., Jiao Y., Zhang Y., Zhu Y., Liu Z., Qiao T., Liu C., Zhang X., Zhou M. (2017). Implantation of Unibody Single-Branched Stent Graft for Patients with Type B Aortic Dissections Involving the Left Subclavian Artery: 1-Year Follow-Up Outcomes. Cardiovasc. Interv. Radiol..

[19] Zhao Z., Qin J., Yin M., Liu G., Liu X., Ye K., Wang R., Shi H., Li W., Jiang M. (2020). In Situ Laser Stent Graft Fenestration of the Left Subclavian Artery during Thoracic Endovascular Repair of Type B Aortic Dissection with Limited Proximal Landing Zones: 5-Year Outcomes. J. Vasc. Interv. Radiol..

[20] Reutersberg B., Gleason T., Desai N., Ehrlich M., Evangelista A., Braverman A., Myrmel T., Chen E.P., Estrera A., Schermerhorn M. (2022). Neurological Event Rates and Associated Risk Factors in Acute Type B Aortic Dissections Treated by Thoracic Aortic Endovascular Repair. J. Thorac. Cardiovasc. Surg..

[21] Buth J., Harris P.L., Hobo R., van Eps R., Cuypers P., Duijm L., Tielbeek X. (2007). Neurologic Complications Associated with Endovascular Repair of Thoracic Aortic Pathology: Incidence and Risk Factors. A Study from the European Collaborators on Stent/Graft Techniques for Aortic Aneurysm Repair (EUROSTAR) Registry. J. Vasc. Surg..

[22] Khoynezhad A., Donayre C.E., Bui H., Kopchok G.E., Walot I., White R.A. (2007). Risk Factors of Neurologic Deficit after Thoracic Aortic Endografting. Ann. Thorac. Surg..

[23] Amabile P., Grisoli D., Giorgi R., Bartoli J.-M., Piquet P. (2008). Incidence and Determinants of Spinal Cord Ischaemia in Stent-Graft Repair of the Thoracic Aorta. Eur. J. Vasc. Endovasc. Surg..

[24] Schlösser F.J.V., Verhagen H.J.M., Lin P.H., Verhoeven E.L.G., van Herwaarden J.A., Moll F.L., Muhs B.E. (2009). TEVAR Following Prior Abdominal Aortic Aneurysm Surgery: Increased Risk of Neurological Deficit. J. Vasc. Surg..

[25] Cooper D.G., Walsh S.R., Sadat U., Noorani A., Hayes P.D., Boyle J.R. (2009). Neurological Complications after Left Subclavian Artery Coverage during Thoracic Endovascular Aortic Repair: A Systematic Review and Meta-Analysis. J. Vasc. Surg..

[26] Matsumura J.S., Rizvi A.Z. (2010). Left Subclavian Artery Revascularization: Society for Vascular Surgery® Practice Guidelines. J. Vasc. Surg..

[27] Rizvi A.Z., Murad M.H., Fairman R.M., Erwin P.J., Montori V.M. (2009). The Effect of Left Subclavian Artery Coverage on Morbidity and Mortality in Patients Undergoing Endovascular Thoracic Aortic Interventions: A Systematic Review and Meta-Analysis. J. Vasc. Surg..

[28] Maldonado T.S., Dexter D., Rockman C.B., Veith F.J., Garg K., Arko F., Bertoni H., Ellozy S., Jordan W., Woo E. (2013). Left Subclavian Artery Coverage during Thoracic Endovascular Aortic Aneurysm Repair Does Not Mandate Revascularization. J. Vasc. Surg..

[29] Howard D.P.J., Banerjee A., Fairhead J.F., Perkins J., Silver L.E., Rothwell P.M. (2013). Population-Based Study of Incidence and Outcome of Acute Aortic Dissection and Premorbid Risk Factor Control Clinical Perspective: 10-Year Results from the Oxford Vascular Study. Circulation.

[30] Pacini D., Di Marco L., Fortuna D., Belotti L.M.B., Gabbieri D., Zussa C., Pigini F., Contini A., Barattoni M.C., De Palma R. (2013). Acute Aortic Dissection: Epidemiology and Outcomes. Int. J. Cardiol..

[31] Piazza M., Squizzato F., Xodo A., Saviane G., Forcella E., Dal Pont C., Grego F., Antonello M. (2021). Determination of Optimal and Safest Proximal Sealing Length During Thoracic Endovascular Aortic Repair. Eur. J. Vasc. Endovasc. Surg..

