# Supplementary material for: Long-Term Outcomes in Thoracic Endovascular Aortic Repair for Complicated Type B Aortic Dissection or Intramural Hematoma Depending on Proximal Landing Zone
Source: J Clin Med. 2023 Aug 18;12(16):5380. doi: 10.3390/jcm12165380 (PMC10455428; doi:10.3390/jcm12165380)
Supplement: Supplementary file 1 [file jcm-12-05380-s001.zip › jcm-2524927-supplementary.pdf]

Supplementary files

Supplementary Table S1: Demographics by healthy landing zone

Characteristic	Total (n=94)	Non-HLZ (n=32)	HLZ (n=62)	*p*-value
Age, years, median (Q1, Q3)	70 (59,78)	67 (59,72)	72 (59,79)	.185
Males	65 (69%)	20 (62%)	45 (73%)	.352
Females	29 (31%)	12 (38%)	17 (27%)	
Body mass index, mean (range)	26 (17-37)	27 (21-37)	26 (17-35)	.173
**Aortic disease**				
Type B aortic dissection	84 (89%)	27 (84%)	57 (92%)	.300
Acute	73	25	48	
Subacute	11	2	9	
Type B intramural hematoma	10 (11%)	5 (16%)	5 (8%)	
Acute	3	2	1	
Subacute	7	3	4	
Initial maximum aortic diameter, millimeter, median (Q1, Q3)	42 (37,52)	42 (39,49)	43 (36,54)	.911
**Comorbidities**				
Arterial Hypertension	57 (80%)	28 (88%)	47 (76%)	.278
Nicotine abuse	31 (33%)	12 (38%)	19 (31%)	.644
Chronic kidney disease	26 (28%)	13 (41%)	13 (21%)	.054
Dyslipidemia	23 (24%)	5 (16%)	18 (29%)	.207
Coronary heart disease	20 (21%)	5 (16%)	15 (24%)	.430
Diabetes mellitus	9 (10%)	4 (12%)	5 (8%)	.484
COPD	7 (7%)	5 (16%)	2 (3%)	.043

COPD=chronic obstructive pulmonary disease; HLZ=healthy landing zone; Q1=quartile 1 (25%); Q3=quartile 3 (75%).

Supplementary Table S2: In-hospital and procedural data by healthy landing zone

**Characteristic**	**Total (n=94)**	**Non-HLZ (n=32)**	**HLZ (n=62)**	***p*-value**
Length of stay, days, median (Q1, Q3)	13 (8,19)	12 (9,19)	14 (8,19)	.755
Time to treatment, days, median (Q1, Q3)	6 (1,13)	8 (2,11)	5 (1,14)	.397
Indication for TEVAR (n (%))				.045
• Pain	18 (19%)	11 (34%)	7 (11%)	
• Hypertension	5 (5%)	2 (6%)	3 (5%)	
• Malperfusion	26 (28%)	8 (25%)	18 (29%)	
• Expansion or Progression	24 (26%)	8 (25%)	16 (26%)	
• Aortic Rupture	21 (22%)	3 (9%)	19 (31%)	
Stent-grafts used for TEVAR	106	34	72	n.a.
• Gore	91	31	60	
• Jotec	8	0	8	
• Cook	5	3	2	
• Medtronic	2	0	2	
Proximal Landing Zone[#]				
• Zone ≤ 2	39 (41%)	8 (25%)	31 (50%)	.020
• Zone ≥ 3	55 (59%)	24 (75%)	31 (50%)	
LSA management				
• Carotid-axillary/subclavian bypass	14 (15%)	3 (9%)	11 (18%)	
• Parallel graft	8 (9%)	2 (6%)	6 (10%)	
• Partial coverage w/o debranching	8 (9%)	0 (0%)	8 (18%)	
• Full coverage w/o debranching	9 (10%)	3 (9%)	6 (10%)	

HLZ=healthy landing zone; Q1=quartile 1 (25%); Q3=quartile 3 (75%); LSA=left subclavian artery; TEVAR=thoracic aortic endovascular repair, n.a.= not applicable. [#] = according to the Ishimaru Zones [8]

Supplementary Table S3: Clinical and morphological outcome by healthy landing zone

Outcome	Total (n=94)	Non-HLZ (n=32)	HLZ (n=62)	*p*-value
Follow-up, months, median (Q1, Q3)	53 (14,111)	49 (14,117)	61 (17,112)	.669
Aortic reintervention, overall	21 (22%)	10 (31%)	11 (29%)	.187
• Distal stent-graft extension	11 (12%)	6 (19%)	5 (8%)	
o Aneurysm growth	10 (11%)	5 (16%)	5 (8%)	
o Aortic rupture	1 (1%)	1 (3%)	0 (0%)	
• Proximal stent-graft extension (EL IA)	9 (10%)	4 (13%)	5 (8%)	
• Relining of type III endoleak	1 (1%)	0 (0%)	1 (2%)	
Aortic growth (≥5mm)	1 (1%)	5 (16%)	7 (11%)	.531
Stroke	5 (5%)	3 (9%)	2 (3%)	.208
Spinal cord ischemia	3 (3%)	0 (0%)	3 (5%)	.200
Stent-graft migration	0 (0%)	0 (0%)	0 (0%)	n.a.
Debranching failure (carotid-subclavian or axillary bypass)	1 (1%)	0 (0%)	1 (2%)	1.00
Retrograde TAAD	5 (5%)	2 (6%)	3 (5%)	1.00
pSINE	6 (6%)	1 (3%)	5 (8%)	.660
30-day mortality	9 (10%)	3 (9%)	6 (10%)	1.00
Overall mortality	42 (45%)	13 (41%)	29 (47%)	.590

*dSINE=distal stent-graft induced new entry; EL IA= type IA endoleak; HLZ=healthy landing zone; Q1=quartile 1 (25%); Q3=quartile 3 (75%); pSINE=proximal stent-graft induced new entry; TAAD=type A aortic dissection, *=statistical significance; n.a.= not applicable*
